# Identification, characterization and expression analysis of passion fruit (*Passiflora edulis*) microRNAs

**DOI:** 10.1007/s13205-019-2000-5

**Published:** 2020-01-02

**Authors:** Sujay Paul, Juan Luis de la Fuente-Jiménez, Camila Garibay Manriquez, Ashutosh Sharma

**Affiliations:** Tecnologico de Monterrey, School of Engineering and Sciences, Centre of Bioengineering, Campus Queretaro, Av. Epigmenio González, No. 500, Fracc. San Pablo, 76130 Querétaro, Mexico

**Keywords:** Passion fruit, microRNA (miRNA), Minimum folding free energy index (MFEI), microRNA expression, miRNA targets

## Abstract

microRNAs (miRNAs) are highly conserved, short (~ 21-nucleotide), endogenous, non-coding RNA molecules that play major roles in post-transcriptional silencing by guiding target mRNA cleavage or translational inhibition. In this study, applying high-stringent genome-wide computational-based approaches, a total of 28 putative miRNAs belonging to 17 miRNA families were identified from an antioxidant-rich medicinal plant passion fruit (*Passiflora edulis*). Inter-tissue (leaves and fruits) and inter-varietal (yellow and purple fruit varieties) quantitative study of six putative passion fruit miRNAs (ped-miR160, ped-miR164, ped-miR166, ped-miR393, ped-miR394, and ped-miR398) showed differential expression. Using psRNATarget tool, a total of 25 potential target proteins of the characterized passion fruit miRNAs were also identified. Most of the target proteins identified in this study, including SQUAMOSA promoter binding, Class III HD-Zip, NAC, Scarecrow, APETALA2, Auxin response factor, MYB, and superoxide dismutase, were found to be involved in development, metabolism, and defense/stress response signaling.

**Electronic supplementary material:**

The online version of this article (10.1007/s13205-019-2000-5) contains supplementary material, which is available to authorized users.

## Introduction

microRNAs (miRNAs) are small (~ 21-nucleotide) non-coding endogenous RNA molecules that play a vital post-transcriptional regulatory role in gene expression by target mRNA cleavage or translational inhibition (Jones-Rhoades et al. 2006). In plant, mature miRNAs are generated from the long stem-loop primary transcript (pre-miRNA) by a dicer-like RNA endonuclease via the intermediate step of pre-miRNA synthesis, and finally the RNA inducing silencing complex (RISC) guided by ARGONAUTE 1 (AGO1) protein directs the miRNA to the complementary target mRNA sequence (Voinnet 2009). Plant microRNAs are reported to possess important functions in several metabolic and biological pathways such as tissue development and differentiation, biotic and abiotic stress responses, phytohormones signaling, and secondary metabolite production (Wu 2013; Rajwanshi et al. 2014; Paul et al. 2011, 2017, 2018; Sharma et al. 2019; Gupta et al. 2017). Nonetheless, the evolutionary highly conserved nature of an extensive number of miRNAs simplified the process of characterization of novel miRNA orthologs in new plant species through homologs identification (Zhang et al. 2006b; Ye et al. 2013; Sharma et al. 2019). However, only the computational-based homology approach for the identification of miRNAs in new plant species can generate false-positive results, and hence several other parameters such as length, minimum free energy (MFE), and the minimum folding free energy index (MFEI) of the pre-miRNAs need to be studied to increase the precision of the prediction (Paul et al. 2018; Sharma et al. 2019). Moreover, experimental validation of the predicted miRNAs is highly recommended (Sharma et al. 2019).

*Passiflora edulis*, commonly known as passion fruit is a flowering vine native to Brazil and cultivated worldwide with distinctive aromas and flavors making it a popular additive to many tropical fruit juice blends (Talcott et al. 2003). The most common varieties of this fruit are with yellow (*P. edulis* var *flavicarpa*) and purple (*P. edulis* var *edulis*) skin (Xiaojing and Liuxin 1994). Both the varieties contain a yellow soft pulp and lots of seeds inside a hard rind (Supplementary file 1) (López-Vargas et al. 2013). The characteristic color of yellow passion fruit is due to provitamin A carotenes and xanthophylls, while the anthocyanins contribute to the spectacular deep purple color to the purple variety (Talcott et al. 2003). Because of their acidic flavor and higher juice yield, yellow passion fruits are most widely used for processed juices, while due to their sweeter taste, purple varieties are typically consumed fresh (Talcott et al. 2003). Passion fruit has recently gained a lot of attention worldwide due to it being a rich source of powerful antioxidants as well as for showing anti-inflammatory, anti-cancer, anti-diabetic, anti-hypertension, and anti-aging properties (Vera et al. 2019; Ayala-Zavala et al. 2011). On the other hand, the peel of passion fruit contains saponin, triterpenoid, flavonoids, and phenolic compounds that can help to inhibit elastase activity facilitating the production of anti-wrinkle cosmetic cream (Vera et al. 2019). Typical phytochemicals found in passion fruit are passiflorine, harmine, harman, harmol, and harmaline (Pereira et al. 2014). Moreover, the leaves of passion fruit also contain several flavonoids such as isoorientin, isovitexin, and vitexin which have been employed in several European and American countries as sedatives and anti-inflammatory agents (Ferreres et al. 2007). Despite the fact that miRNAs regulate several metabolic processes in plants, none of the miRNA-related studies have been performed so far in passion fruit, and none are listed in the miRBase. It has been confirmed that several conserved, as well as species-specific miRNAs, are very important for plants in different biological processes and hence, profiling miRNAs in a non-model plant is essential to understand the regulation of various biological phenomena. With the recent draft genome sequence available (Wu et al. 2019), the current study offers an insight of miRNAs and their respective targets in passion fruit using bioinformatics as well as wet-lab approach for better understanding the physiological processes in this plant. In summary, in this study for the first time, we have generated the miRNA and respective target profile of passion fruit and performed a comparative miRNA expression study between vegetative and reproductive tissues (leaves and fruits) as well as between two different varieties (yellow and purple skins).

## Materials and methods

### Computational prediction of miRNA

For the in silico prediction of potential passion fruit miRNAs, a reference set of mature plant miRNAs were retrieved from miRBase database (http://www.mirbase.org/cgi-bin/sequence_get.pl) and aligned against the whole genome sequence of passion fruit. The reference set consisted of a total 1370 mature miRNA sequences including *Arabidopsis thaliana* (428 mature sequences), *Glycine max* (756 mature sequences), and *Vitis vinifera* (186 mature sequences). BLASTn tool was used for the alignment and sequences with exact matches were chosen manually. The possible precursor (pre-miRNA) sequences of approximately 400 nucleotides (nt) (200 nt upstream and 200 nt downstream to the BLAST hit region) were mined and sequences coded for proteins were eliminated. To check the reliability of the possible precursors, secondary structures were predicted using MFOLD (http://unafold.rna.albany.edu/?q=mfold) webserver. Since an authentic secondary structure of the precursor is considered as one of the vital factors to be a miRNA candidate, some previously demonstrated strict filtering criteria were applied during prediction, such as: (1) the precursors must form a stem‐loop structure containing mature miRNA sequences within one arm, (2) the potential miRNA sequences should not be positioned at the terminal loop of the hairpin structures, (3) mature miRNAs should have fewer than nine mismatches with the opposite miRNA*sequence, and (4) the predicted secondary structures must have low MFE and high MFEI values, since it is required for distinguishing the miRNAs from other RNAs molecules (MFEIs of tRNAs, rRNAs or mRNAs candidates are 0.64, 0.59 and 0.62–0.66, respectively) (Zhang et al. 2006a). The MFE or ΔG (−kcal/mol) values generated from the MFOLD web server of the stem-loop structures were used for calculating the MFEI values using the following formula:

$${\text{MFEI}} = \, \frac{{\left( {\text{MFE/length of RNA sequence}} \right)\, \times \,100}}{{{\text{\% GC content}}}}$$

### Prediction of miRNA targets

The precise or near-precise complementarity of plant miRNAs and their targets facilitated in silico miRNA target prediction in non-model plants. In this study, web tool psRNATarget (http://plantgrn.noble.org/psRNATarget/?dowhat=Help) was used to identify the potential miRNA targets of passion fruit. Due to unavailability of passion fruit protein database in the psRNATarget web server, target search was conducted against the protein database of *Populus trichocarpa*, which is considered as the evolutionary closest species of passion fruit (Wu et al. 2019). The parameters were adjusted manually, such as maximum expectation value of 3, translation inhibition ranges of 9–11 nt, number of top targets of 10, penalty for G:U pair of 0.5, and number of mismatches allowed in seed region of 1.5.

### Collection of plant materials, RNA extraction, and miRNA expression analysis

To validate the predicted results, fresh ripe yellow and purple passion fruits and leaves were collected from the local field in Queretaro, Mexico. Total RNA including small RNA was extracted from leaves and both the fruit varieties using the miRNeasy Mini Kit (Qiagen) and pooled separately for each sample. The quality and quantity of RNA samples were measured with Nanodrop One (Thermo Scientific), and subsequently polyadenylated (using modified oligo dT primer) as well as reverse transcribed using mRQ Buffer (2 ×) and enzyme provided with Mir-X miRNA First-Stand Synthesis kit (Takara, Tokyo, Japan). The resulting cDNA was then amplified by T100 Thermal Cycler (Bio-Rad, CA, USA) using the entire predicted miRNA sequence as forward primer and the adapter-specific mRQ3′ primer provided with Mir-X miRNA qRT-PCR TB Green Kit (Takara, Tokyo, Japan) as the reverse primer. Selected six passion fruit miRNAs (miR160, miR164, miR166, miR393, miR394, and miR398) were experimentally validated in this study. The PCR was programmed as follows: initial denaturation at 94 °C for 3 min followed by 45 cycles of denaturation at 94 °C for 30 s and annealing at 60 °C for 30 s, extension at 72 °C for 25 s, and a final elongation step at 72 °C for 7 min. The resulted PCR products (~ 80 bp) were checked in 2% agarose gel. To study the quantitative differential expression pattern of the aforesaid passion fruit miRNAs between fruits and leaves as well as between two varieties, a quantitative real-time PCR (qPCR) was performed. The reaction was made in 12.5 μl volume containing: 3.8 μl of ddH_2_O, 6.25 μl of TB Green Advantage Premix (2 ×), 0.23 μl of ROX Dye (50 ×), 0.1 μl of miRNA-specific primer (10 μM), 0.1 μl of mRQ3′ primer (10 μM), and 2 μl of cDNA. Each sample was done with three technical replicates. Step One Real-Time PCR System (Applied Biosystems, Carlsbad, CA) and Mir-X miRNA TB Green qRT-PCR kit (Takara, Tokyo, Japan) were used, respectively for the qPCR experiment. The qPCR was programmed as follows: initial denaturation at 95 °C for 10 s followed by 45 cycles of denaturation at 95°C for 5 s and annealing at 60 °C for 20 s, dissociation curve at 95 °C for 30 s, 55 °C for 20 s, and 95 °C for 20 s. The detail of miRNA-specific primers used in this study is presented in Supplementary file 1. The comparative Ct (2^−ΔΔCT^) method was employed to determine the relative fold changes and U6 was employed as an internal reference for the miRNA qPCR analysis (Livak and Schmittgen 2001).

## Results and discussions

### Characterization of passion fruit miRNAs

**Fig. 1:**
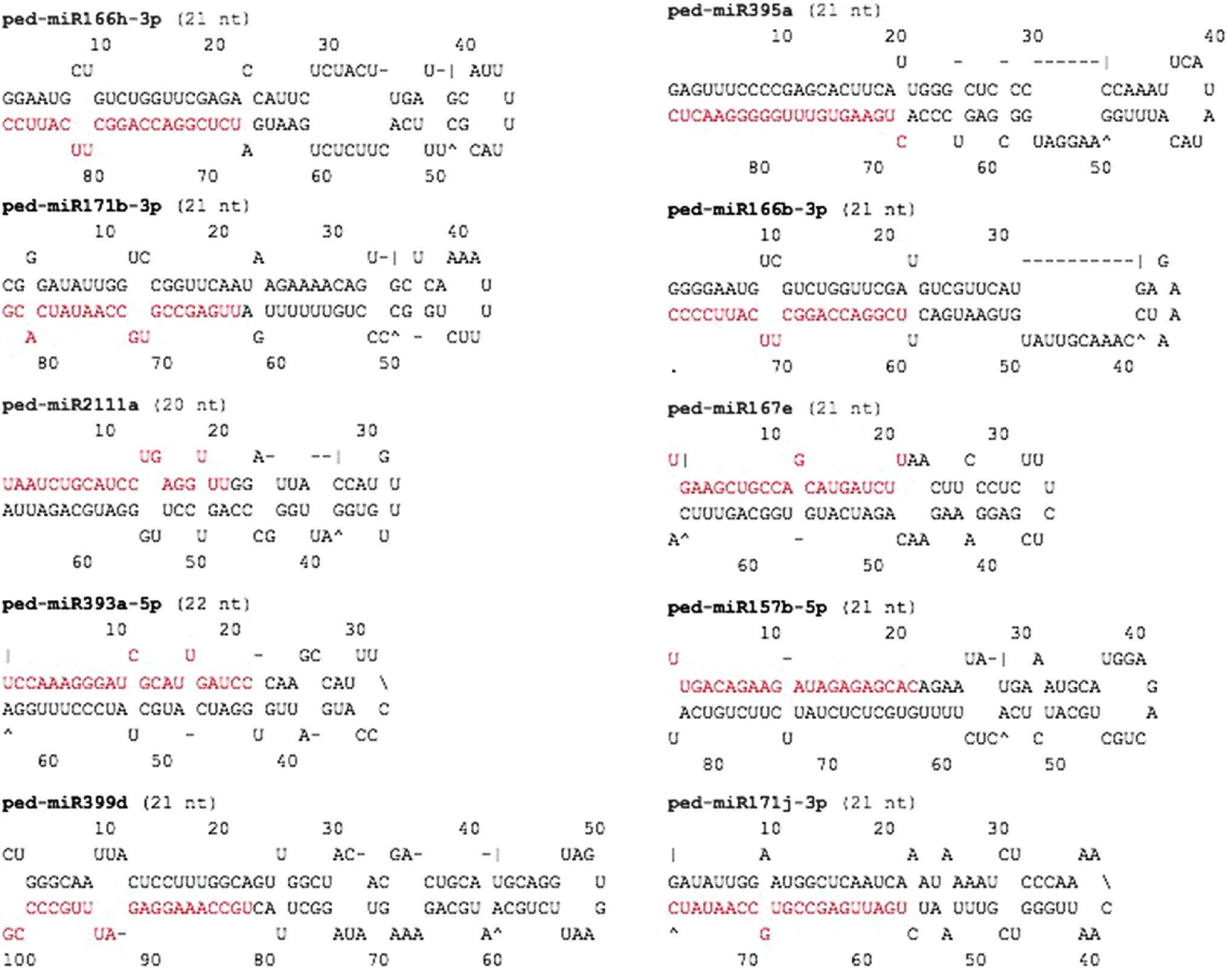
Secondary stem-loop structures of the predicted passion fruit miRNA precursors/pre-miRNAs. Respective miRNAs are represented with red font

**Fig. 2:**
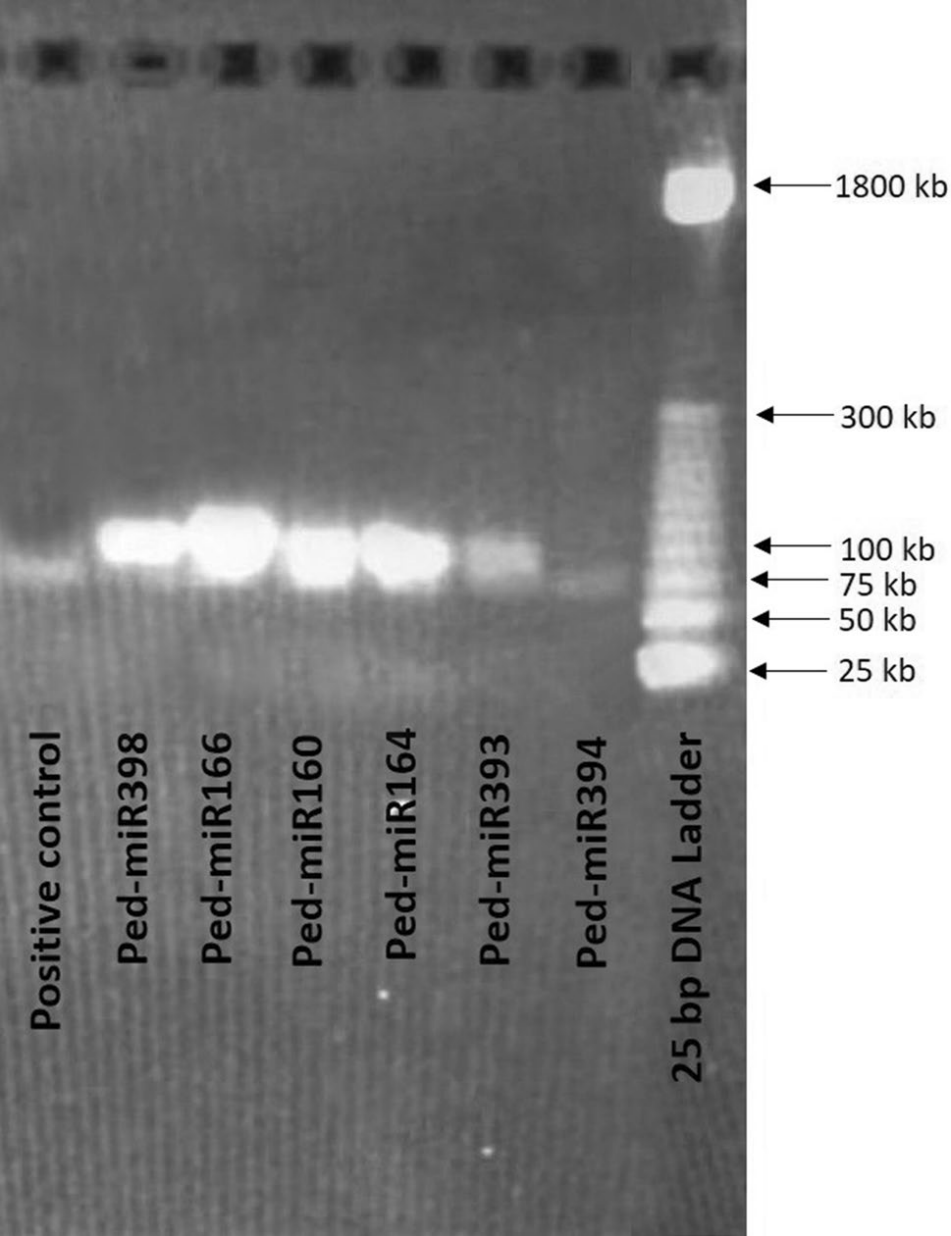
Validation of selected passion fruit miRNAs (ped-miR160, ped-miR164, ped-miR166, ped-miR393, ped-miR394, and ped-miR398) by semiquantitative reverse transcription PCR (fruit tissues). The resulting PCR products were checked in 2% agarose gel with EtBr staining. U6 was employed as a positive control

Employing rigorous filtering approach, 28 conserved passion fruit miRNAs were identified in this study belonging to 17 miRNA families (miR156/miR157, miR160, miR162, miR164, miR166, miR167, miR169, miR171, miR172, miR319, miR393, miR394, miR395, miR397, miR398, miR399, and miR828) (Table 1). The majority of the identified passion fruit miRNAs are 21 nt long. The length of the predicted pre-miRNA varied from 60 nt to 185 nt with an average length of 102 nt. In addition, all the predicted pre-miRNAs produced secondary structures with stem loop and 50% of the mature miRNAs were found either at the 5′ or 3′ end corroborating Gorodkin et al. (2006) studies which exhibit an equal distribution of miRNAs in both arms in other plant species. Moreover, 75% of the predicted passion fruit miRNAs started with the nucleotide uracil (U) agreeing with the study of Zhang et al. (2008) that miRNA-mediated regulation is greatly dependent on the uracil present at the first position of the mature miRNA. The GC content of passion fruit miRNAs had an average of 46.25%. It is well established that low MFE values of stem-loop precursors attain more stable predictions (Bonnet et al. 2004); in this study, the MFE values of the precursors ranged from − 24.80 to − 79.40 with an average of − 44.02, while the MFEI values oscillated between 0.70 and 1.30 with an average of 0.95, excluding the possibility of being other small RNAs. Furthermore, as exhibited in Table 1, clusters of miRNA genes in passion fruit are abundant. Several clusters are compacted indicating some groups of miRNAs are expressed from specific transcription elements (polycistron) such as miR166a-5p, miR166b-3p, miR166h-3p, miR166h-5p; miR171b-3p, miR171j-3p, miR171k-5p; miR319b, miR319I, miR319p; miR399d, miR399e, and miR399i. However, in the current analysis, the maximum number of miRNA members was found to be present in miR166 family. The predicted secondary structures of passion fruit miRNA precursors with higher MFEI values (top 10) are shown in Fig. 1. The in silico prediction of passion fruit miRNAs was found accurate, as all the selected miRNAs displayed expected bands in the agarose gel (around ~ 80 bp) (Fig. 2).

**Table 1:** Summary of the identified miRNAs from passion fruit

Identified miRNAs	LM (nt)	Query miRNAs	miRNA sequences	Accession	Location	LP (nt)	GC %	MFEs (ΔG)	MFEI
ped-miR156a-5p	20	ath-miR156a-5p	UGACAGAAGAGAGUGAGCAC	MUZT01071036.1	5′	82	47.56	– 51.10	1.31
ped-miR157a-5p	21	ath-miR157a-5p	UUGACAGAAGAUAGAGAGCAC	MUZT01093399.1	5′	84	40.48	– 42.90	1.26
ped-miR160c-5p	21	ath-miR160c-5p	UGCCUGGCUCCCUGUAUGCCA	MUZT01072383.1	5′	79	58.23	– 43.50	0.94
ped-miR162a	22	ath-miR162a-5p	UGGAGGCAGCGGUUCAUCGAUC	MUZT01067906.1	5′	81	48.15	– 28.50	0.73
ped-miR164b-5p	21	ath-miR164b-5p	UGGAGAAGCAGGGCACGUGCA	MUZT01048418.1	3′	195	44.10	– 60.40	0.70
ped-miR166a-5p	21	gma-miR166a-5p	GGAAUGUUGUCUGGCUCGAGG	MUZT01089702.1	5′	131	45.80	– 56.80	0.94
ped-miR166b-3p	21	ath-miR166b-3p	UCGGACCAGGCUUCAUUCCCC	MUZT01068357.1	3′	80	48.75	– 44.90	1.15
ped-miR166h-3p	21	gma-miR166h-3p	UCUCGGACCAGGCUUCAUUCC	MUZT01172784.1	3′	88	45.45	– 39.20	0.98
ped-miR166h-5p	21	gma-miR166h-5p	GGAAUGUUGUUUGGCUCGAGG	MUZT01069089.1	5′	125	37.60	– 44.50	0.94
ped-miR167e	21	gma-miR167e	UGAAGCUGCCAGCAUGAUCUU	MUZT01041392.1	5′	67	44.78	– 35.80	1.19
ped-miR169a	21	gma-miR169a	CAGCCAAGGAUGACUUGCCGG	MUZT01219929.1	5′	179	43.58	– 66.30	0.84
ped-miR169b	21	gma-miR169b	CAGCCAAGGAUGACUUGCCGA	MUZT01009503.1	5′	99	46.46	– 39.40	0.85
ped-miR171b-3p	21	ath-miR171b-3p	UUGAGCCGUGCCAAUAUCACG	MUZT01091311.1	3′	84	44.05	– 38.40	1.03
ped-miR171j-3p	21	gma-miR171j-3p	UGAUUGAGCCGUGCCAAUAUC	MUZT01028930.1	3′	77	37.66	– 37.80	1.30
ped-miR171k-5p	21	gma-miR171k-5p	CGAUGUUGGUGAGGUUCAAUC	MUZT01137815.1	5′	79	46.84	– 29.10	0.78
ped-miR172b	21	vvi-miR172b	UGAAUCUUGAUGAUGCUACAC	MUZT01049317.1	3′	120	47.50	– 42.20	0.74
ped-miR319b	21	ath-miR319b	UUGGACUGAAGGGAGCUCCCU	MUZT01035900.1	3′	171	46.20	– 74.80	0.94
ped-miR319l	22	gma-miR319l	UUGGACUGAAGGGAGCUCCUUC	MUZT01112275.1	3′	185	46.49	– 79.40	0.92
ped-miR319p	21	gma-miR319p	UUUUGGACUGAAGGGAGCUCC	MUZT01179548.1	3′	76	47.37	– 32.80	0.91
ped-miR393a-5p	22	ath-miR393a-5p	UCCAAAGGGAUCGCAUUGAUCC	MUZT01078953.1	5′	64	46.88	– 33.10	1.10
ped-miR394a-5p	20	gma-miR394a-5p	UUGGCAUUCUGUCCACCUCC	MUZT01119677.1	5′	78	50.00	– 33.40	0.85
ped-miR395a	21	gma-miR395a	CUGAAGUGUUUGGGGGAACUC	MUZT01200730.1	3′	88	51.14	– 50.20	1.11
ped-miR397a	21	ath-miR397a	UCAUUGAGUGCAGCGUUGAUG	MUZT01096698.1	5′	79	51.90	– 39.50	0.96
ped-miR398d	21	gma-miR398d	UGUGUUCUCAGGUCGCCCCUG	MUZT01004242.1	3′	102	50.00	– 42.30	0.82
ped-miR399d	21	ath-miR399d	UGCCAAAGGAGAUUUGCCCCG	MUZT01051618.1	3′	102	49.02	– 55.30	1.10
ped-miR399e	21	gma-miR399e	UGCCAAAGGAGAUUUGCCCAG	MUZT01038065.1	3′	102	45.10	– 35.80	0.77
ped-miR399i	21	gma-miR399i	UGCCAAAGGAGAAUUGCCCUG	MUZT01092893.1	5′	60	45.00	– 24.80	0.91
ped-miR828b	22	gma-miR828b	UCUUGCUCAAAUGAGUAUUCCA	MUZT01146558.1	5′	123	39.84	– 42.70	0.87

*LM* length of mature miRNAs, *LP* length of precursor

### Identification of potential target transcripts of putative passion fruit microRNAs

In this study, a total of 25 possible target transcripts of passion fruit miRNAs were identified including some uncharacterized proteins and among those potential targets, several were found to be participating in signaling and metabolic pathways, defense mechanisms/stress response signaling, and cellular development (Table 2). Other targets were implicated in metal ion binding, ATP binding, DNA and RNA binding, and symporter activities. Therefore, target genes can be divided into three different groups: metabolism-related targets, stress-responsive targets, and transcription factors. However, the same miRNAs can have several distinct functions such as development as well as stress response signaling. Some miRNA: target pairs are well conserved among different plants species such as (1) transcription factors HD-ZIPs, participated in a variety of processes during plant growth and development, are principally targeted by miR166 family in poplar, rice, apple, and *Arabidopsis*; (2) Transcription factors NO APICAL MERISTEM (NAM) and NAC which were found to be involved in shoot development, fruit ripening, and also flavonoid biosynthesis are mostly targeted by miR164 family (Morishita et al. 2009; Zeng et al. 2015); (3) F-Box proteins, these are involved in many plants’ vegetative and reproductive growth and development are targeted by both miR393 and miR394 families in *Arabidopsis*, poplar, rice, and apple; (4) Auxin is a key regulator of virtually every aspect of plant growth and development from embryogenesis to senescence and auxin response factors (ARFs) which control the auxin-responsive genes as well as stress-responsive signals are often targeted by miR160 families; (5) superoxide dismutases (SODs), the major antioxidant defense systems in plants are the primary target of miRNA398 family in various plant species (Ye et al. 2013; Wang et al. 2004; Lu et al. 2005; Archak and Nagaraju 2007; Zhang et al. 2009; Colaiacovo et al. 2010; Sunkar et al. 2012; Bouzroud et al. 2018). Although few other miRNAs: target pairs are also conserved among different plant species in this study, we selected the six very important aforesaid miRNAs for the qPCR-based quantitative expression analysis.

**Table 2:** Potential targets of identified passion fruit miRNAs

miR family	Name of the target transcript	Molecular function	Biological process
miR157	SQUAMOSA promoter binding-like protein	DNA-binding transcription factor, metal ion binding	Regulation of transcription
miR160	Auxin response factor	DNA binding	Auxin-activated signaling pathway, leaf senescence, negative regulation of transcription, flavonoid biosynthesis
miR164	NAC domain protein	DNA binding	Regulation of transcription, defense response, flavonoid biosynthesis
miR166	Class III HD-zip protein	DNA and lipid binding	Development of shoot apical meristem
	AG-motif binding protein-2	Sequence-specific DNA and zinc ion binding	Regulation of transcription
	Chaperone protein ClpB3	ATP binding	Protein metabolic process and protein refolding
miR169	Nuclear transcript factor Y subunit A	DNA-binding, protein heterodimerization activity	Abscisic and gibberellic acid signaling pathway, regulation of gene expression, positive regulation of photomorphogenesis
miR171	Serine-rich protein	RNA binding	mRNA cis splicing via spliceosome
	Scarecrow-like protein 6	Sequence-specific DNA binding	Regulation of transcription
	Ethylene-responsive transcription factor RAP2-7	DNA binding	Ethylene-activated signaling pathway
	Floral homeotic protein APETALA2	DNA binding	Flower, seed, and plant ovule development, cell differentiation
	Transcription factor AHAP2	DNA binding	Not found
	Inositol phosphate kinase	ATP and metal ion binding	Defense response, lateral root development
	Transcription factor GAMYB-like	Sequence-specific DNA binding	Anther development, cell, and pollen sperm cell differentiation, regulation of transcription, response to ethylene
miR393	Auxin response factor	DNA binding	Auxin-activated signaling pathway, leaf senescence, negative regulation of transcription
	Protein transport inhibitor response 1	Auxin binding and auxin receptor activity	Cell cycle, auxin activated and ethylene signaling pathway, defense response, response to auxin
miR394	F-box only protein	Photoreceptor activity	Circadian rhythm. Flower development, protein ubiquitination
miR395	ATP-sulfurylase	ATP binding	Response to cytokinin and cadmium ion, sulfate assimilation
	Sulfate adenylyltransferase	Nucleotide binding	Purine ribonucleotide and sulfur compound metabolic process
miR397	Laccase	Copper ion binding	Lignin biosynthetic and catabolic pathway
miR398	Superoxide dismutase	Copper ion binding	Cellular response to light intensity, oxidative stress, ozone, and salt stress
	Protein LAS1	Terpenes’ synthase activity and magnesium ion binding	Defense response and metabolic process
	Dissimilatory sulfite reductase beta subunit	Metal ion binding	Sulfur compound metabolic process
	Zinc metalloprotease EGY2	Metallopeptidase activity	Not found
miR828	Myb-like transcription factor	Sequence-specific DNA binding	Circadian regulation, histone H acetylation, photoperiodism, response to auxin, ethylene, abscisic acid, gibberellin, jasmonic acid, and salt stress

### Expression analysis of selected passion fruit miRNAs

**Fig. 3:**
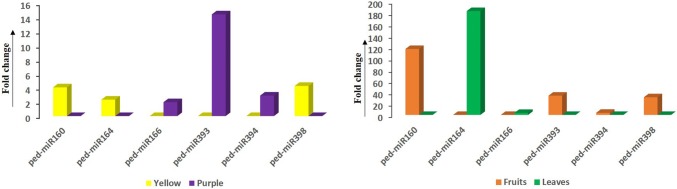
Graphical representation of differential expression pattern (fold change) of selected passion fruit miRNAs (ped-miR160, ped-miR164, ped-miR166, ped-miR393, ped-miR394, and ped-miR398) between vegetative and reproductive tissues (leaves and fruits) as well as between two different varieties (yellow and purple). U6 was employed as an internal reference

The qRT-PCR results showed a differential expression pattern of the selected miRNAs between fruits and leaves (both from yellow varieties) as well as between two different fruit varieties. The expression of miR160, and miR164, and miR398 was found to be higher in the yellow fruit variety than the purple one with a fold change of 4.05, 2.32, and 4.25, respectively. However, in the purple fruit variety miRNAs, miR166, miR393, and miR394 were found to be upregulated compared to the yellow one with the respective fold change of 1.96, 14.35, and 2.89. Furthermore, the miRNAs miR160, miR393, miR394, and miR398 showed higher expression in the fruit compared to leaves with a fold change of 116.16, 33.94, 3.77, and 31.12, respectively, while only miR164 and miR166 were found to be overexpressed in the leaves with a fold change of 183.01 and 3.53, respectively, compared to fruits. The fold change can be explained since the overexpression of certain miRNAs is linked to a decrease in the target transcript expression in the tissue and vice versa (Neutelings et al. 2012). For example, auxin is essential for plant development and high levels of auxin can repress the expression of the MYB–bHLH–WD (MBW) complex and thereby suppress the biosynthesis of anthocyanin. On the other hand, ARFs, target transcripts of miR-160 can control the expression of auxin-inducible genes by binding to auxin response elements in their promoters and therefore indirectly regulate the anthocyanin biosynthesis (Sun et al. 2017). Similarly, a relation between the overproduction of the NAC domain transcription factor (target of miR-164) and anthocyanin accumulation was recorded in blood‐fleshed peaches (Zhou et al. 2015). In this study, low expression of both ped-miR160 and miR-164 in the purple variety as compared to yellow variety indicated the accumulation of anthocyanin in purple variety and thus corroborates with the previous reports. Nevertheless, this differential expression pattern of passion fruit microRNAs obtained from the qPCR data demonstrates that a cell can control the target expression/functions in certain types of tissues or samples by manipulating the miRNA expression (Farh et al. 2005). The results of the qRT-PCR-based differential expression of the current study are shown in Fig. 3.

## Conclusion

To the best of our knowledge, this is the first report of the characterization of passion fruit microRNAs and their targets. In this report using strict filtering criteria, a total of 28 conserved passion fruit miRNAs belonging to 17 miRNA families as well as 25 corresponding targets were computationally identified. Among the predicted passion fruit miRNAs, six selected miRNAs (ped-miR160, ped-miR164, ped-miR166, ped-miR393, ped-miR394, and ped-miR398) were validated by semiquantitative RT-PCR and their quantitative expression was measured by qPCR in leaves and fruits as well as between two different fruit varieties. All the aforesaid miRNAs displayed significantly differential expression between the samples. Among the predicted passion fruit miRNA targets, several were found to be involved in metabolism, defense/stress response signaling, development, and flavonoid biosynthesis. Nevertheless, the identification of miRNAs and their targets is a key step towards the initiation of the miRNA-related study in a non-model plant and we believe that our current study will be helpful for strengthening the research on miRNA-mediated regulation in herbal plants.

## Electronic supplementary material

Below is the link to the electronic supplementary material.

Supplementary material 1 (DOCX 178 kb)
