# Supplementary material for: Identification, characterization and expression analysis of passion fruit (Passiflora edulis) microRNAs
Source: 3 Biotech. 2020 Jan 2;10(1):25. doi: 10.1007/s13205-019-2000-5 (PMC6938847; doi:10.1007/s13205-019-2000-5)
Supplement: Supplementary file 1 — Supplementary material 1 (DOCX 178 kb) [file 13205_2019_2000_MOESM1_ESM.docx]

**Table.** Detail of the primers used in this study

| **Primer Name** | **Sequence** |
| --- | --- |
| miR160 | 5’-TGCCTGGCTCCCTGTATGCCA-3’ |
| miR164 | 5’-TGGAGAAGCAGGGCACGTGCA-3’ |
| miR166 | 5’-TCGGACCAGGCTTCATTCCCC-3’ |
| miR393 | 5’-TCCAAAGGGATCGCATTGATCC-3’ |
| miR394 | 5’-TTGGCATTCTGTCCACCTCC-3’ |
| miR398 | 5’-TGTGTTCTCAGGTCGCCCCTG-3’ |

**Figure .** Yellow (*P. edulis* var *flavicarpa*) and purple (*P. edulis* var *edulis*) passion fruit varieties.

**
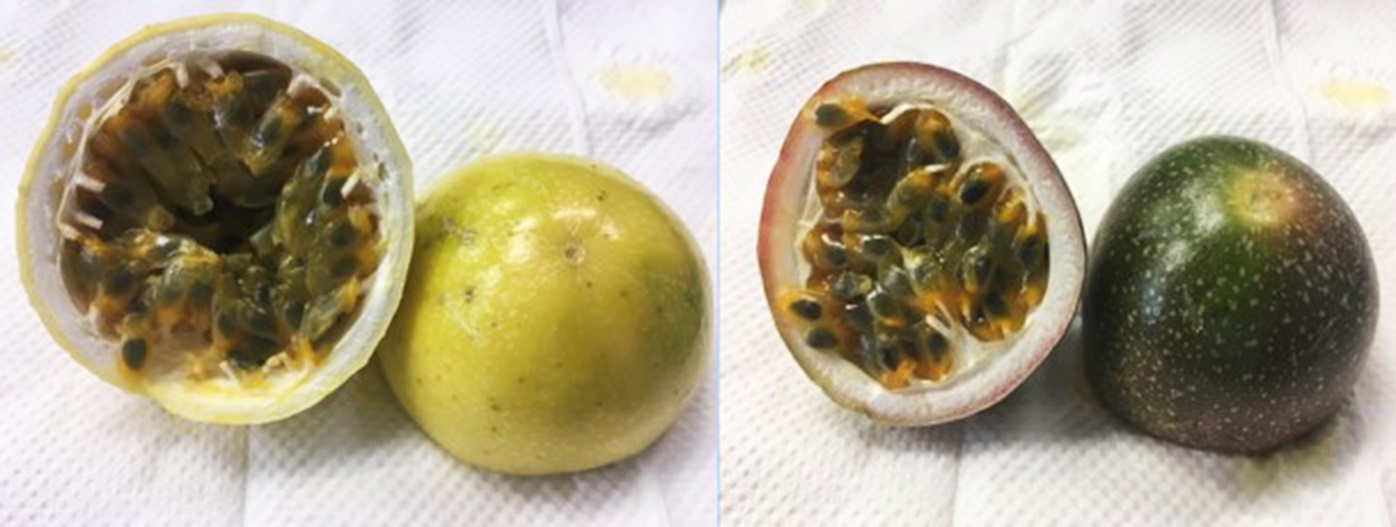
**
